# Bongkrekic Acid and Its Novel Isomers: Separation, Identification, and Determination in Food Matrices

**DOI:** 10.3390/toxins17050223

**Published:** 2025-05-02

**Authors:** Suhe Dong, Danli Liu, Runfeng Lin, Yingjie Zhu, Peihong Zhu, Xin Jiang, Jie Mao, Yanqing Cao, Jing Peng, Tianyue Zhao, Danning Shen, Tao Li, Kun He, Na Wang

**Affiliations:** National Center of Biomedical Analysis, Beijing 100039, Chinatli@ncba.ac.cn (T.L.)

**Keywords:** bongkrekic acid, cis–trans isomer, biotoxin, UHPLC-MS/MS, food matrix, *Burkholderia gladioli*

## Abstract

The toxicity associated with bongkrekic acid (BKA) is severe due to its chemical structure, which also facilitates high mortality rates; however, its isomer, isobongkrekic acid (iBKA), with only minor structural variance, demonstrates marked differences in toxicity. This discrepancy in structural properties and toxicity highlights that risks have been potentially underestimated within current detection standards for BKAs. In this study, a novel BKA trans isomer at the C8 and C9 double carbon bonds (E-configuration), termed iBKA-neo, was successfully separated and identified. Subsequently, the multiple reaction monitoring parameters and chromatographic conditions for three BKA isomers were optimized, enabling effective separation within 15 min via UHPLC-MS/MS, among which the ammonium positive adduct ions yielded significantly higher response intensities for all BKA isomers than traditional deprotonated molecules. Additionally, distinct differences in the ion ratios between iBKA-neo and BKA were utilized for preliminary screening. On this basis, the extraction and enrichment strategies for BKAs were optimized in food matrices and validated comprehensively with good linearity (0.25–500 μg/kg), a superior limit of quantification (0.25 μg/kg), acceptable recoveries (82.32–114.84%), and stable intraday and interday precision (an RSD less than 12.67%). These findings significantly contribute to ecotoxicology and the formulation of safety standards concerning BKAs.

## 1. Introduction

The rise in bongkrekic acid (BKA) poisoning incidents globally has become a pressing concern, often linked to improper food preparation and storage, which leads to *Burkholderia gladioli* proliferation—primarily affecting foods such as *Tremella fuciformis* and wet rice dumping noodles [1,2,3]. With over 2000 reported cases worldwide leading to more than 3000 individuals affected, the death toll has reached approximately 1200 people—a mortality rate of 40–60%. These incidents are not uniformly distributed; East Asia bears a disproportionate burden, with more than 90% of all BKA poisonings [1,3]. This regional clustering is linked to dietary preferences favoring rice and noodles, particularly fermented forms such as rice dumplings and sour noodles, for which the humid fermentation process creates an ideal environment for *Burkholderia gladioli* [3,4,5]. The toxicity and lethality of BKA have prompted food safety regulators across several countries to establish detection methods and guidelines [6,7].

The mechanism by which BKA triggers its toxic effects is through intricate biochemical interactions with mitochondrial function, which attributes to BKA’s chemical structure—a polyunsaturated long-chain fatty acid with three carboxyl groups—making it weakly acidic and capable of producing isomers during its synthesis by *Burkholderia gladioli*, differing only in the geometric configuration around double bonds [8,9]. Specifically, BKA binds to ATP/ADP transporter protein (ANT), a critical component of the inner mitochondrial membrane [9,10], preventing the normal function of the transport process, leading to energy depletion, cellular dysfunction, and even cell death [10]. Adding another layer of complexity, the cis‒trans isomerization at the double carbon bonds within BKA alters its conformation and thus influences binding affinity with proteins such as ANT [11]. Despite these minor structural variances, they can significantly affect target specificity and toxicity among different BKA isomers [12,13]. Research in mice has shown that isobongkrekic acid (iBKA), a trans isomer of BKA, results in only one-fifth of the toxicity of BKA when tested in vivo [14]. This finding highlights that risks have been potentially underestimated within current detection standards for BKA and its variants.

To date, only one BKA isomer, iBKA, featuring a trans double carbon bond at C20 and C21, has been widely documented [15]. However, the presence of seven such bonds in BKA suggests that more potential isomers await discovery. Analytical chemists have successfully distinguished iBKA from its parent compound through various techniques, but identifying and quantifying other BKA isomers remains challenging due to their similar chromatographic behavior [14]. The limitations of LC-MS in BKA analysis stem from its reliance on the retention time and mass-to-charge ratio for isomer differentiation. Given BKAs’ identical molecular weights and minimal polarity variations, co-elution interference in complex biological matrices frequently compromises separation efficiency and introduces substantial quantification errors [16,17]. Practical challenges persist, including intricate sample pretreatment procedures, pronounced matrix effects, and the absence of standardized extraction protocols across diverse sample matrices, all contributing to elevated false-positive rates in food detection [18]. While high-resolution mass spectrometry (HRMS) has demonstrated potential in aflatoxin isomer analysis through sub-ppm mass accuracy and MS/MS fragment ion resolution [19,20], its current application to BKAs remains restricted to pure standards rather than real biological samples. Notably, recent advancements utilizing HPLC-Orbitrap HRMS technology coupled with a Fe_3_O_4_/HNT-based rapid extraction procedure have achieved the successful detection of BKA and iBKA in rice matrices, marking progress in practical implementation [7]. To ensure comprehensive monitoring and prevention strategies against BKA poisoning incidents, it is essential to develop robust analytical methods capable of detecting all known and unknown isomers present in food samples. Such advancements will not only improve public health surveillance but also contribute valuable insights into the biochemistry underlying this lethal toxin’s mode of action.

In this study, we identified a novel BKA isomer—designated iBKA-neo—in *Burkholderia gladioli* fermentation broth via chromatographic separation and successfully performed its structural elucidation through nuclear magnetic resonance (NMR). Further, we developed a UHPLC-MS/MS detection scheme to enable the absolute quantitative detection of three BKA isomers across multiple foods susceptible to *Burkholderia gladioli* contamination. Notably, the ammonium positive adduct ions yielded significantly higher response intensities for all BKAs than traditional deprotonated molecules, achieving a superior detection sensitivity. Moreover, a preliminary screening strategy to distinguish iBKA-neo and BKA was performed using distinct differences in ion ratios. These findings significantly contribute to the formulation of safety standards concerning BKAs.

## 2. Results and Discussion

### 2.1. Structural Identification of iBKA-Neo

In an effort to mimic real-world sample analysis, we endeavored to detect BKA using *Tremella fuciformis* infected with *Burkholderia gladioli* as a model system. Leveraging established literature protocols [21], we preprocessed *Tremella fuciformis* to extract and enrich BKA and its isomer. However, when employing a mature UHPLC-MS/MS detection strategy for the determination of the absolute content of the BKAs, an evident shoulder peak emerged at the original retention time position of the BKAs (Figure S1A). Notably, this shoulder peak did not correspond to BKA or its known isomer iBKA. Through iterative adjustments to the mobile-phase gradient, prolonged elution times, and gradual increases in organic-phase proportions, we successfully resolved the aforementioned shoulder peaks from BKA (Figure S1B). Significantly, our findings suggested the potential separation and identification of a novel BKA isomer distinct from both BKA and iBKA.

**Figure 1:**
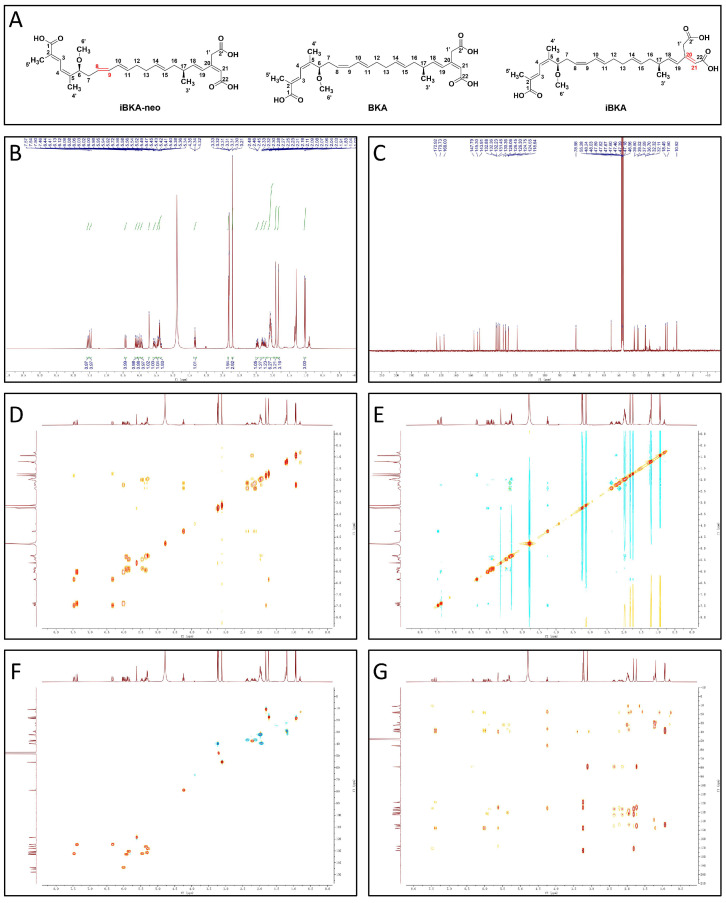
**The structural identification of iBKA-neo.** (**A**) The structural formulas of BKA, iBKA, and iBKA-neo (the double carbon bonds marked in red represent the cis–trans isomeric position). (**B**) ^1^H NMR of iBKA-neo (400 MHz, CD_3_OD). (**C**) ^13^C NMR of iBKA-neo (101 MHz, CD_3_OD). (**D**) ^1^H-^1^H DQF-COSY of iBKA-neo (F1, 3424.7 Hz; F2, 3424.7 Hz, CD_3_OD). (**E**) ^1^H-^1^H NOESY of iBKA-neo (F1, 3424.7 Hz; F2, 3424.7 Hz, CD_3_OD). (**F**) ^1^H-^13^C HSQC of iBKA-neo (^1^H 3424.7 Hz, ^13^C 16,602.4 Hz). (**G**) ^1^H-^13^C HMBC of iBKA-neo (^1^H 3268.0 Hz, ^13^C 22,137.0 Hz). In detail, ^1^H NMR (400 MHz, CD_3_OD): δ 7.56 (d, J = 12.0 Hz, 1H), 7.48 (d, J = 16.1 Hz, 1H), 6.42 (d, J = 12.2 Hz, 1H), 5.95 (dd, J = 14.6, 10.3 Hz, 1H), 6.11 (dd, J = 16.1, 7.5 Hz, 1H), 6.03 (dd, J = 14.7, 10.3 Hz, 1H), 5.72 (s, 1H), 5.56 (dt, J = 14.6, 6.4 Hz, 1H), 5.42–5.33 (m, 1H), 5.42–5.33 (m, 1H), and 5.46 (dd, J =14.7, 7.3 Hz, 1H). 3.33 (s, 2H), 3.21 (s, 3H), 4.34 (t, J = 7.0 Hz, 1H), 2.46 (dt, J = 13.5, 7.0 Hz, 1H), 2.21–2.15 (m, 1H), 2.37–2.27 (m, 1H), 2.14–2.01 (m, 2H), 2.14–2.01 (m, 2H), 2.14–2.01 (m, 2H), 1.91 (s, 3H), 1.83 (s, 3H), 1.03 (d, J = 6.7 Hz, 3H). ^13^C NMR (101 MHz, CD_3_OD): δ 132.36, 124.75, 124.55, 130.36, 143.91, 132.88, 118.64, 132.23, 128.06, 131.46, 126.45, 39.80, 55.28, 78.98, 36.70, 37.59, 39.52, 32.32, 32.11, 10.92, 17.50, 18.45, 172.92, 168.00.

Further, *Burkholderia gladioli* was utilized for fermentation. After 14 days, the fermentation broth was collected and enriched. The separation and preparation of BKA isomers in fermentation broth were conducted via silicone column and preparative chromatography and the components of the extraction mixture were purified to obtain BKA isomers with a single property (Figure 1A). Finally, the structure of the single-component BKA isomer was confirmed by ^1^H NMR (Figure 1B and Figure S1C) and ^13^C NMR (Figure 1C and Figure S1D), and the positional relationships of H-H, C-C, and C-H were confirmed by two-dimensional (2D) NMR (Figure 1D–G and Figure S1E–H). After peak assignment and the matching of the C-H positions, an isomer structure of BKA distinct from those previously reported was isolated from the bacterial fermentation broth (Figure 1D–G and Figure S1E–H) [15]. Compared with the reported structural information of BKA, the chemical shifts and coupling constants at positions C8 and C9 exhibited significant variations, and ^1^H NMR showed different splitting forms, whereas the chemical shifts and coupling constants at other positions showed no obvious changes, which could indicate that they are cis/trans isomers at double carbon bonds C8 and C9 (Figure 1B,C and Figure S1C,D). The newly discovered BKA isomer at positions C8 and C9 has the following ^1^H NMR (400 MHz, CD_3_OD): δ 6.03 (dd, J = 14.7, 10.3 Hz, 1H), 5.46 (dd, J = 14.7, 7.3 Hz, 1H), and ^13^C NMR (101 MHz, CD_3_OD): δ 132.88, 126.45, which is in the trans configuration, and it was named iBKA-neo (*E*-configuration). The corresponding BKA has the following ^1^H NMR (600 MHz, CD_3_OD): δ 5.98 (t, J = 10.9 Hz, 1H), 5.21 (dt, J = 10.9, 7.6 Hz, 1H), and ^13^C NMR (150 MHz, CD_3_OD): δ 130.55, 123.73, which is in the cis configuration (*Z*-configuration) (Figure 1B,C and Figure S1C,D). iBKA, in contrast, is a trans isomer of BKA at the C20 and C21 double carbon bonds (*E*-configuration) [15].

The polyketide synthase (PKS) encoded by Bon is implicated in the biosynthetic pathway of BKA in *Burkholderia gladioli* [3]. The distinct functions of Bon PKS are correlated with various stages of BKA biosynthesis, including the elongation of the polyketide fatty chain, the formation of β-polyketide, and chain branching events [3]. Notably, the cis or trans elimination occurring during β-branching may be responsible for the emergence of *E/Z* isomeric forms of BKA and iBKA. Previous studies have employed multi-module trans AT-PKS to dissect the complex assembly process of BKA polymers, thereby predicting the relative and absolute configurations of BKA based on PKS [22]. Interestingly, computational simulations have suggested there are *E*-double carbon bonds at C8 and C9, whereas the observed absolute conformation of BKA (*Z*-configuration) diverges from the predicted *E*-configuration, exhibiting a distinct deviation in the geometry of the double bond [22]. However, our newly discovered iBKA-neo is consistent with the absolute conformation simulated by the calculation (*E*-configuration), thereby validating the predictability and authenticity of the existence of iBKA-neo.

### 2.2. Characteristics of Mass Spectrometry for BKA Isomers

**Figure 2:**
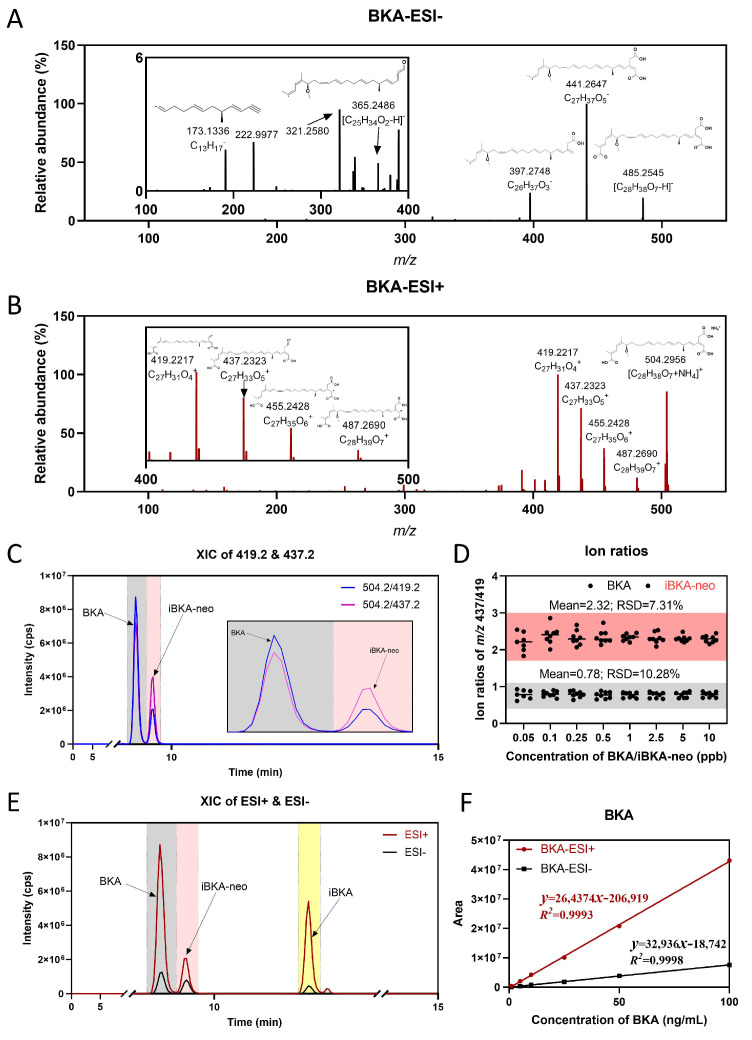
**Mass spectrometry fragmentation patterns and detection mode characteristics of BKA isomers.** (**A**) Fragment ion characteristics of BKA in ESI negative mode [M-H]^−^, high-resolution mass spectrometry in SIM-ddMS2 detection mode, and HCD (%) steps of 5, 10, 15, and 20 for normalization. (**B**) Fragment ion characteristics of BKA in ESI positive mode [M+NH_4_]^+^, high-resolution mass spectrometry in SIM-ddMS2 detection mode, with HCD steps of 5, 10, 15, and 20 for normalization. (**C**) Extraction ion chromatograms (XIC) of fragment ions at *m/z* 419 and *m/z* 437 of BKA and iBKA-neo in ESI positive mode. (**D**) The ion ratios of fragments at *m/z* 437 to *m/z* 419 for BKA and iBKA-neo in ESI positive mode. (**E**) MS response total ion chromatogram (TIC) of BKA isomers with the same concentration detected in ESI positive and negative mode. (**F**) Linear range of the MS response detected by BKA in ESI positive and negative mode.

We aimed to delineate the fragmentation patterns of BKA isomers via HRMS. Owing to the nature of BKAs as long-chain fatty acid compounds with weak acidity, existing detection methods typically favor negative ion mode for mass spectrometric analysis [7]. To address this trend, we explored the fragmentation patterns under such conditions and established an SIM-ddMS2 mass spectrometry detection scheme utilizing *m/z* 485 as the parent ion. Each isomer displayed prominent product fragments at *m/z* 441, *m/z* 397, *m/z* 365, *m/z* 321, and *m/z* 173 (Figure 2A and Figure S2A,B). The structure and fragmentation mechanism of these product fragments in ESI negative mode were elucidated via MS Frontiers software (Version 8.1) (Figure S2C). However, we encountered a limitation, as the product ion response intensities for different BKA isomers were indistinguishable (Figure 2A and Figure S2A,B).

In an effort to overcome this limitation, we expanded our investigation into ESI positive mode [M+NH_4_]^+^, observing high responses at *m/z* 504 for all BKA isomers. The SIM-ddMS2 detection scheme using *m/z* 504 as the parent ion yielded significant product fragments at *m/z* 487, *m/z* 455, *m/z* 437, and *m/z* 419 (Figure 2B and Figure S3A,B). The response intensity distribution patterns were nearly identical for BKA and iBKA, except for iBKA-neo, which exhibited a distinct ion ratio between *m/z* 437 and *m/z* 419 product fragments, likely attributable to its unique chemical structure. High-sensitivity mass spectrometry was performed to further validate the observed differential response in product fragment distribution between BKA and iBKA-neo. The results confirmed a greater ion ratio of *m/z* 437 to *m/z* 419 for iBKA-neo than for BKA (Figure 2C). By utilizing the ion ratio at *m/z* 437 to *m/z* 419 as a distinguishing factor, we observed that BKA maintained a relatively stable ion ratio of 0.78 with an RSD of 10.28%, whereas the initial ion ratio of iBKA-neo was 2.32, with an RSD of 7.31% (Figure 2D). This marked difference in the ion ratio between BKA and iBKA-neo substantiates the utility of high-sensitivity mass spectrometry in differentiating isomers.

Furthermore, optimized qualitative and quantitative mass spectrometry transmissions for detecting BKA and its isomers were subsequently developed on Triple Quad 7500. This included the optimization of acquisition and fragmentation parameters, resulting in an MRM list specific to these compounds (Table S2). Interestingly, at equivalent concentrations, the ESI positive mode resulted in significantly higher response intensities for BKAs than did the ESI negative mode across a wide concentration range from 1 to 100 ng/mL (Figure 2E,F and Figure S4A,B). This indicates that ESI positive mode has better efficiency for BKA isomers detection, both in response intensity and detection specificity.

### 2.3. Optimized Chromatography for Distinguishing BKA Isomers

The separation of BKA and its isomers, particularly the newly identified iBKA-neo, poses significant challenges in analytical chemistry because of their similar chromatographic behavior [23]. Early studies established foundational separation techniques using thin-layer chromatography (TLC) and column chromatography, though these methods lacked resolution for distinguishing isomers with subtle stereochemical differences [24]. Recent advances in hyphenated LC-MS/MS platforms have improved sensitivity and specificity, enabling the simultaneous quantification of BKAs in complex matrices like fermented foods and biological samples [14,25]. Notably, chiral stationary-phase HPLC coupled with HRMS now resolves critical isomer pairs, revealing significant differences in toxicity profiles tied to spatial configurations [26]. This necessitates the development of novel chromatographic techniques capable of effectively separating these compounds for accurate quantification.

#### 2.3.1. ACQUITY UPLC BEH C18 Column Optimization

**Figure 3:**
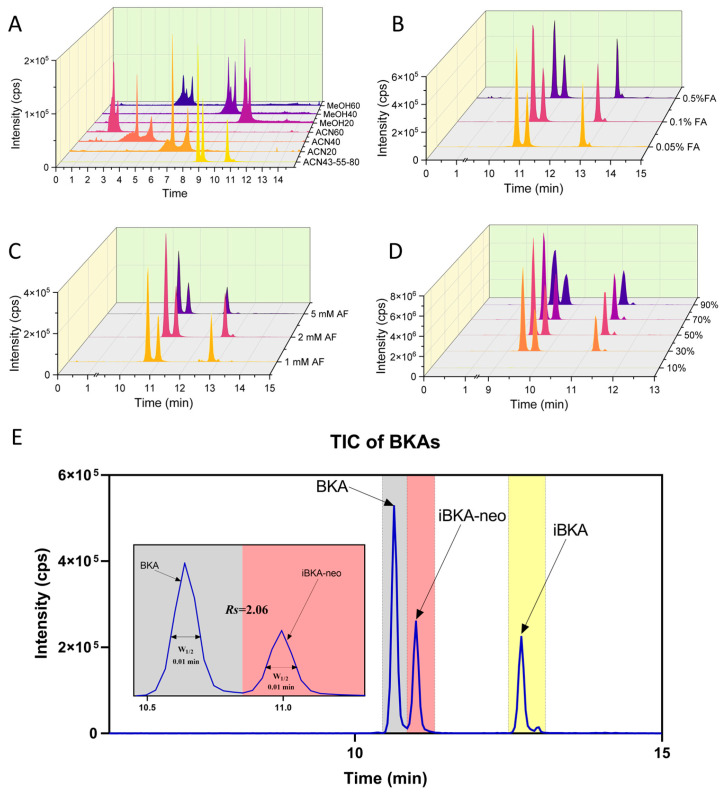
**Optimization of the chromatographic separation method for BKA isomers (BEH C18 column).** (**A**) TICs of MEOH and ACN as organic phases with initial mobile phases of 20%, 40%, and 60% and ACN segmented gradient changes of 43%, 55%, and 80% for the separation efficiency of BKA isomers. (**B**) TICs of the separation efficiency of different proportions of FA (0.05%, 0.1%, 0.5%) on BKA isomers in the mobile phase. (**C**) TICs of the separation efficiency of different concentrations of AF (1 mM, 2 mM, and 5 mM) on BKA isomers in the mobile phase. (**D**) TICs of the separation efficiency of BKA isomers when different ratios of ACN (10%, 30%, 50%, 70%, and 90%) were used as dilution solvents. (**E**) TIC of the separation efficiency of BKA isomers optimized by chromatographic conditions. W_1/2_ represents the full width at half maximum; *Rs* represents the separation degree.

Initially, investigations were conducted via an ACQUITY UPLC BEH C18 column, which employed MEOH and ACN, owing to their different elution capabilities [27]. Compared with MEOH, ACN was found to exhibit superior elution for BKA, with a low initial ACN concentration, yielding better separation outcomes (Figure 3A). Gradually increasing the ACN proportion facilitated the separation of all three BKA isomers to almost baseline. The role of organic acids and salts in the mobile phase was then explored. While an enhanced mass spectrometry response was observed with higher FA concentrations, this response also led to increased noise signal intensity (Figure 3B). Conversely, increasing the AF concentration slightly improved BKA/iBKA-neo separation but significantly reduced isomer detection responses (Figure 3C). The impact of the ACN concentration as dilution solvents of BKA isomers was also assessed, revealing that increasing the ACN concentration initially increased the MS response before decreasing it, with the RT remaining stable but the peak shapes widening (Figure 3D). Based on these findings, an optimized protocol using ACN as the organic phase with the addition of 0.1% FA and 2 mM AF, 50% ACN as the dilution solvent, and segmented gradient liquid chromatography was established, achieving the effective separation of BKA isomers within 15 min with a resolution of 2.06 between BKA and iBKA-neo (Figure 3E and Table S3).

#### 2.3.2. Exploring Alternative Columns

To assess the potential for enhanced separation capabilities, various column types, including ACQUITY UPLC T3, ACQUITY UPLC Amide, and chiral columns, were tested. The polybutylene terephthalate (PBT) column has emerged as a promising alternative owing to its ability to distinguish compounds based on unsaturation levels and isomerism through π electron interactions [28]. To optimize the PBT column chromatographic parameters, MEOH was selected over ACN for a better separation efficiency of BKA/iBKA-neo at lower concentrations (Figure S5A). Interestingly, an alkaline mobile-phase pH resulted in superior separation compared with acidic conditions, with AB outperforming AF as a buffer salt addition (Figure S5B). However, higher AB concentrations compromised the BKA/iBKA-neo separation, particularly at 5 mM, where isomer peak identification became challenging (Figure S5C). The final PBT column chromatography parameters were set to MEOH with 1 mM AB and segmented gradient liquid chromatography, achieving the effective separation of all three BKA isomers within 13 min and a resolution (Rs) of 1.67 between BKA and iBKA-neo (Figure S5D, Table S4). Although the ACQUITY UPLC BEH C18 column outperformed the PBT column in terms of separation efficiency for these compounds, the latter provided an alternative method for chromatographic separation under alkaline conditions, broadening the analytical toolkit available for BKA isomer analysis.

### 2.4. Optimized Extraction and Enrichment of BKA Isomers in Food Matrices

After establishing robust UHPLC‒MS/MS quantitative detection methods for three BKA isomers, our focus shifted to assessing the method’s applicability within multiple complex food matrices. Nowadays, challenges persist in rapid on-site screening due to matrix interference and low analyte concentrations, prompting innovations in aptamer-based biosensors and simplified extraction protocols [29]. In this study, we chose improperly stored fresh *Tremella fuciformis* as a test case due to its high detectable levels of BKAs [30]. This decision allowed us to refine extraction and enrichment processes specifically tailored to food environments.

**Figure 4:**
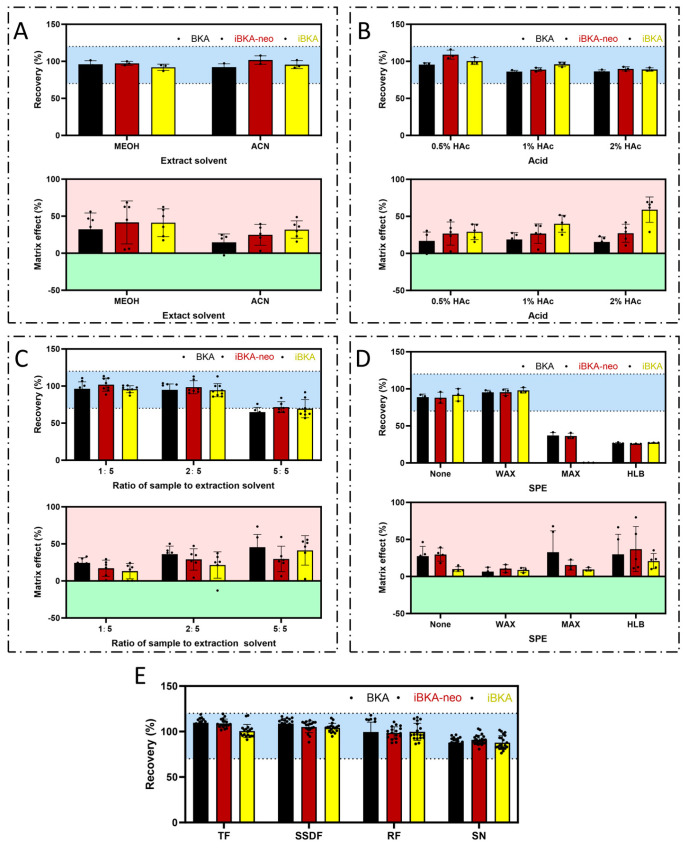
**Optimization of extraction and enrichment methods for BKA isomers.** (**A**) The influence of MEOH and ACN as extraction reagents on the extraction recovery rate and matrix effect of BKA isomers. (**B**) Effects of different ratios of HAc (0.5%, 1%, and 2%) in the extraction reagent on the extraction recovery rates and matrix effects of BKA isomers. (**C**) Effects of the compatibility ratio (1:5, 2:5, or 5:5) of the matrix sample (g) and extraction reagent (mL) on the extraction recovery rates and matrix effects of BKA isomers. (**D**) The influence of different types of SPE columns (WAX, MAX, HLB) on the extraction recovery rates and matrix effects of BKA isomers. (**E**) The optimized extraction and enrichment method was used to determine the recovery rates of BKA isomers in different food matrices. The blue area represents the acceptable range of the recovery rate of 70–120%, the red area represents the signal enhancement of the matrix effect, and the green area represents the signal suppression of the matrix effect.

First, both MEOH and ACN were effective at extracting BKA isomers, with recovery rates ranging from 70% to 120% (Figure 4A). However, ACN offered a less pronounced matrix enhancement effect than MEOH did (Figure 4A). Second, the addition of HAc improved the BKA isomers’ recovery rates across multiple concentrations (0.5%, 1%, and 5%). The addition of 1% HAc provided an optimal recovery rate in line with the target range while minimizing matrix effects (Figure 4B). In an analogous vein, only a 0.5% concentration of NH_3_·H_2_O achieved the desired recovery rate of 70–120% (Figure S6A) but induced a more pronounced matrix enhancing effect compared to HAc (Figure S6A). Then, there was a systematic decrease in the recovery rates for all BKA isomers as the proportion of the food matrix increased. Notably, at the highest examined compatibility ratio of 5:5 (g:mL), the recovery rates remained outside the optimal range of 70–120%, falling short of attaining the desired analytical performance (Figure 4C). Moreover, an augmentation in the food matrix proportion corresponded with a pronounced increase in the matrix-enhancing effect. Further, among the WAX, MAX, and HLB SPE columns, only WAX had a beneficial effect on the BKA isomers’ recoveries without exceeding the acceptable matrix enhancement (Figure 4D). Moreover, increasing the ultrasonication time from 10 to 30 min increased the recovery rates closer to 100%, with no significant change in the matrix effects across all the tested times (10, 20, and 30 min) (Figure S6B).

Finally, our optimized method for extracting BKA isomers included the use of ACN as the extraction solvent at a compatibility ratio of 1:5 of the food matrix (g) to the extraction solvent (mL). The addition of 1% HAc facilitates effective extraction, which is then enhanced through 10 min of ultrasonic treatment. This approach was successful in achieving recovery rates between 82.32% and 114.84% for BKA isomers across multiple food matrices (*Tremella fuciformis*, sweet soup dumpling flour, rice flour, and sour noodles) (Figure 4E), with the stipulation that a WAX-SPE column must be used specifically for sour noodles to maintain these standards (Figure S6C). This refined methodology not only adheres to academic citation and originality requirements but also significantly enhances the specificity and sensitivity of BKA isomer detection in multiple food matrices.

### 2.5. Methodological Validation of BKA Detection in Food Matrices

The validation process for the detection methodology of BKA isomers was carried out meticulously to ensure reliability and accuracy across various food matrices. This section details the selectivity, linearity, sensitivity, recovery, precision, and matrix effect considerations.

***Selectivity.*** A total of 24 blank samples with the same MS patterns were detected to determine if any signals at the same RT as the BKA isomers were present. To address specificity concerns among BKA isomers due to shared fragment ion information, unique quantitative transmissions were selected for each isomer optimized with a distinct collision energy (CE): 12 V for BKA (*m/z* 437), 11 V for iBKA (*m/z* 437), and 15 V for iBKA-neo (*m/z* 419) (Table S2), and the ion ratios at *m/z* 437/419 of each BKA in different matrices were measured to evaluate their consistency (Figure 2D). This optimization facilitated the precise differentiation of these isomers across different food matrices through chromatographic separation, ensuring specific detection without cross-reactivity.

**Figure 5:**
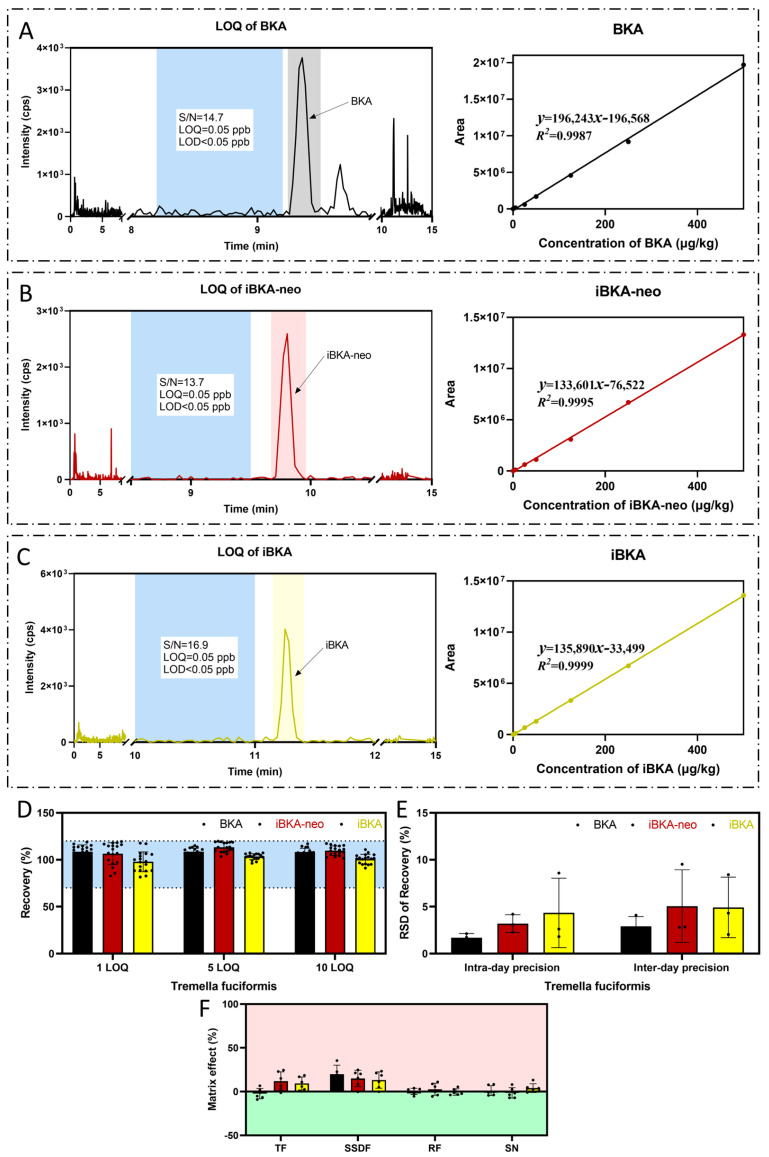
**Methodological validation (*Tremella fuciformis* matrix).** In the *Tremella fuciformis* matrix, the signal-to-noise ratio, LOQ, LOD (left), linear range, and correlation coefficient (right) of BKA (**A**), iBKA-neo (**B**), and iBKA (**C**) are shown. The blue area represents the data source of the noise when confirming the signal-to-noise ratio. (**D**) The recovery rates of BKA isomers at the LOQ, 5 LOQ, and 10 LOQ in the *Tremella fuciformis* matrix. The blue area represents the acceptable range of recovery rates from 70% to 120%. (**E**) The intraday and interday variations in the LOQ, 5 LOQ, and 10 LOQ concentrations of BKA isomers in food matrices. (**F**) The matrix effect in determination of BKA, iBKA, and iBKA-neo in *Tremella fuciformis* matrix.

***Linearity.*** Linearity was assessed by calibrating BKA isomers over a wide concentration range from 0.05 to 100 ng/mL (ppb). All three BKA isomers demonstrated good linearity across this range. For the *Tremella fuciformis* matrix, BKA, iBKA-neo, and iBKA presented *R^2^* values of 0.9987, 0.9995, and 0.9999, respectively, over a 0.05–100 ppb wide concentration range (Figure 5A–C).

***Sensitivity (LOD and LOQ).*** Sensitivity was determined by spiking food matrices with BKA isomers at varying concentrations to evaluate detection and quantification limits based on recovery rates within the acceptable threshold of 70–120% and an RSD ≤ 20% [31]. In all the tested matrices—*Tremella fuciformis*, sweet soup dumpling flour, rice flour, and sour noodles—the LOQ for all three BKA isomers was 0.25 μg/kg (Figure 5A–C), with a 0.08 μg/kg LOD (Figure 5A–C, Table 1).

***Recovery.*** The accuracy of the method was established by spiking blank food matrix samples (*Tremella fuciformis*, sweet soup dumpling flour, rice flour, and sour noodles) with mixed standard solutions containing BKA isomers at concentrations of 0.25, 1.25, and 2.5 μg/kg. The recovery rates for all three BKA isomers across different food matrices were within the acceptable range (70–120%). For example, in *Tremella fuciformis*, the BKA recovery ranged from 106.13% to 114.35%, iBKA recovery ranged from 94.64% to 105.18%, and iBKA-neo recovery ranged from 105.27% to 112.56% (Figure 5D, Table 1).

***Precision.*** The precision evaluation involved assessing the intraday and interday variations in the LOQ (0.25 μg/kg), 5 LOQ (1.25 μg/kg), and 10 LOQ (2.5 μg/kg) concentrations of the BKA isomers in food matrices. The RSD values for both intra- and interday precision were less than 20% across all tested concentration levels in all the matrices (Figure 5E, Table 1).

***The matrix effect.*** The impact of the matrix effect was significant for all BKA isomers at the LOQ concentration. In particular, BKA (−10% to 5%), iBKA-neo (−2% to 20%), and iBKA (2% to 15%) in *Tremella fuciformis* displayed substantial matrix enhancement effects (Figure 5F). To address this challenge, we employed a matrix-matched calibration approach that successfully negated the matrix effect. The implementation of this method allowed us to achieve acceptable analyte recovery (70−120%). This result indicates that matrix matching is essential for the accurate quantification of BKA, iBKA, and iBKA-neo in four food matrices.

### 2.6. Application of the UHPLC‒MS/MS Method for BKA Isomers

Following the comprehensive validation of the UHPLC‒MS/MS method for BKA isomers within food matrices, we sought to apply this strategy to real-world samples. The chosen substrate was inoculated with *Burkholderia gladioli*, which is known for its capacity to produce BKAs [32], and underwent fermentation under controlled laboratory conditions for 14 days.

**Figure 6:**
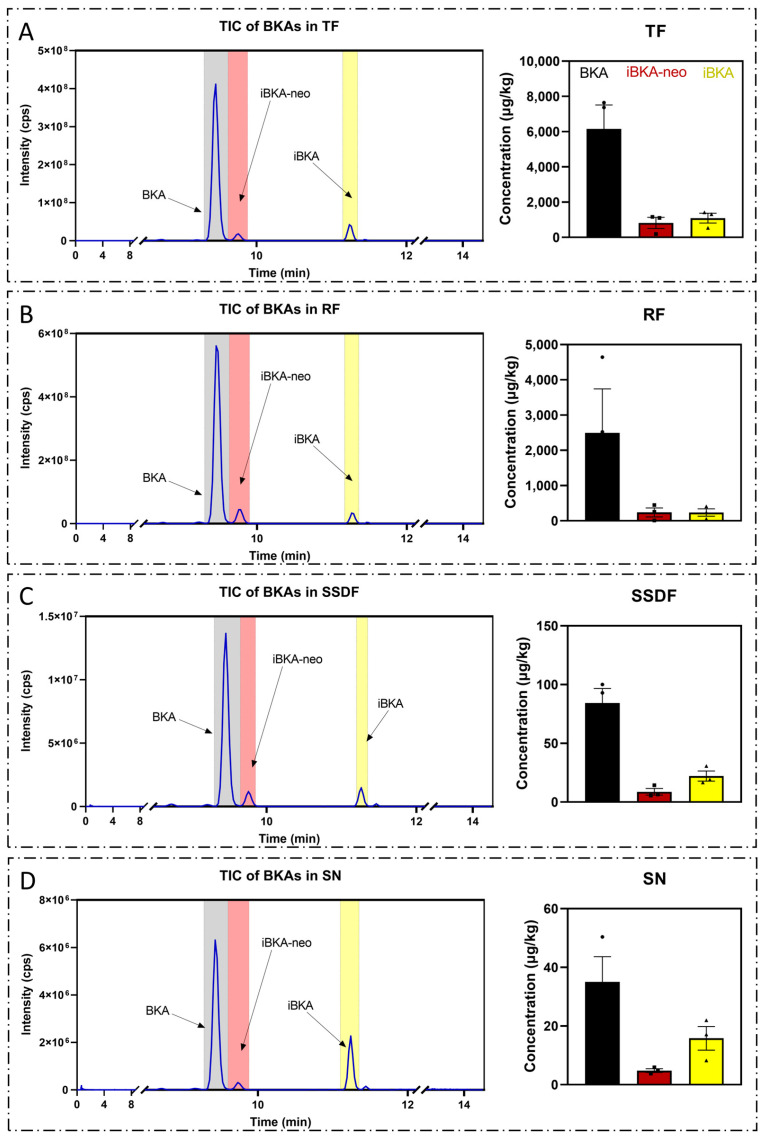
**Application of the UHPLC‒MS/MS method in real-world samples.** (**A**) *Tremella fuciformis*, (**B**) rice flour, (**C**) sweet soup dumpling flour, and (**D**) sour noodles (n = 3, respectively) were tested as fermentation media, and *Burkholderia gladioli* of the same source was inoculated. After 14 days of fermentation, according to the extraction and enrichment methods provided in this study, the accurate content of each BKA isomer in the sample was detected, and the TICs of BKA isomers detected in different food matrices were generated.

Postfermentation, the food matrices were subjected to an extraction and enrichment process as previously optimized during method development. The absolute concentrations of BKA isomers in these real-world samples were subsequently quantified via our refined UHPLC−MS/MS scheme. A detailed analysis revealed that *Tremella fuciformis* (TF, n = 3) presented the highest concentrations: 6158.092 μg/kg for BKA, 820.313 μg/kg for iBKA-neo, and 1090.582 μg/kg for iBKA (Figure 6A). The rice flour (RF, n = 3) displayed significantly higher BKA isomers levels: 2494.289 μg/kg for BKA, 238.224 μg/kg for iBKA-neo, and 235.329 μg/kg for iBKA (Figure 6B). In contrast, the sweet soup dumpling flour (SSDF, n = 3) only contained a BKA concentration of 84.296 μg/kg, an iBKA-neo concentration of 8.748 μg/kg, and an iBKA concentration of 22.141 μg/kg (Figure 6C). Similarly, sour noodles (SN, n = 3) were found to have a BKA concentration of just 35.004 μg/kg, an iBKA-neo concentration of 4.796 μg/kg, and an iBKA concentration of 15.804 μg/kg (Figure 6D).

## 3. Conclusions

In summary, our research has made significant strides in the understanding and analysis of *Burkholderia gladioli*-derived BKA isomers. By leveraging advanced chromatographic separation techniques, we successfully isolated a previously unidentified BKA isomer. Further scrutiny via NMR revealed its unique chemical structure: a trans isomer at the C8 and C9 double carbon bonds (*E*-configuration). This novel finding has been designated as iBKA-neo.

Our investigation extended to HRMS, whereby we uncovered an additional ion mode [M+NH_4_]^+^ for BKA isomers, enhancing the specificity and sensitivity of detection. Through a comprehensive analysis, we delineated the fragmentation patterns characteristic of BKA, facilitating a more detailed understanding of their chemical behavior.

Building on this foundation, our study has devised a robust quantitative analytical strategy for BKA isomers utilizing UHPLC-MS/MS. This method, combined with optimized enrichment and extraction strategies, was meticulously refined to discern various BKA isomers in complex food matrices. Consequently, we have achieved precise separation, a higher sensitivity, and the quantification of BKAs within multiple food matrices. Moreover, this strategy retains the possibility of being extended to other unknown BKA isomers’ separation and determination.

The comprehensive approach we have developed not only represents a significant advancement in analytical chemistry but also provides critical insights for the revision of standard testing methods and formulation of limit standards concerning BKA and its isomers. Our findings highlight the necessity of updating food safety regulations with isomer-specific limits and prioritizing isomer monitoring in high-risk regions. Future work will focus on in vivo toxicity assessments of individual isomers to further inform risk management strategies.

## 4. Materials and Methods

### 4.1. Materials and Reagents

Standard solutions (100 μg/mL) of BKA isomers were purchased from Alta Scientific Co., Ltd. (Tianjin, China). Detailed information on each standard is available in Table S1. The working standard solution (1 μg/mL) contained individual standard solutions diluted 50:50 and mixed with various volumes of acetonitrile (ACN) and water. These standard solutions were stored in the dark at −20 °C for at least one month and diluted accordingly before analysis. *Burkholderia gladioli* (IKCC 20220345) was gifted by IniKem BioPharmaTech Co., Ltd. (Qingdao, China).

Water, ACN and methanol (MEOH) of LC‒MS grade, and ammonium formate (AF) of analytical purity were supplied by Merck (Darmstadt, Germany). Formic acid (FA), acetic acid (HAc), ammonium bicarbonate (AB), and ammonia liquor (NH_3_·H_2_O) were obtained from Sinopharm Group Chemical Reagent Co., Ltd. (Shanghai, China). The ACQUITY UPLC BEH C18 column (2.1 mm ×100 mm, 1.7 μm), Oasis^®^ WAX (1 cc, 30 mg), MAX (3 cc, 60 mg), and PRiME HLB (3 cc, 60 mg) cartridges were obtained from Waters (Milford, MA, USA). The DCpak PBT column (3.0 mm × 150 mm, 3 μm) was purchased from Daicel Corporation (Shanghai, China). The 0.22 μm nylon syringe filter membranes were purchased from SHIMADZU Corporation.

### 4.2. Equipment

An ultra-high-performance liquid chromatography−tandem triple quadrupole mass spectrometer (AB Sciex Triple Quad 7500, AB SCIEX, Singapore City, Singapore) was used for qualitative and quantitative analysis. An AVANCE NEO 400 M spectrometer (Bruker, Switzerland) and an HPLC-Orbitrap Exploris 480 (Thermo Fisher Scientific, Bremen, Germany) were used for the structural identification of the BKAs. An ultrasonic cleaning machine (Lumiere Tech Ltd., Beijing, China) was used to extract the compounds from the samples. A 5424R centrifuge (Eppendorf) and a high-speed refrigerated centrifuge (Thermo Fisher, Germany) were used for the centrifugation of the sample mixture. A vacuum freezing centrifugal concentrator (LABCONCO, America) was used for sample drying. An IKA^®^ Vortex (Germany) and a multichannel vortex (Tuohe Electromechanical Technology, Shanghai, China) were used to vortex mix the sample mixture. An analytical balance (ME204, METTLER TOLEDO) was used to accurately determine the sample mass. A QuSEL^®^ parallel homogenizer (E0301, Alta Scientiffc, China) was used to blend the samples. A positive pressure solid-phase extractor (J2 Scientific PPM48) was used for the solid-phase extraction process.

### 4.3. Bacterial Fermentation and Purification of BKA Isomers

*Burkholderia gladioli* was activated and cultured at 28 °C for 2–3 days. Use an inoculation ring to scrape 2 rings and transfer them to a seed containing medium. Cultivate this at 28 °C and 180 r/min for 48 h. Inoculate 5 mL of aforementioned solution into the fermentation medium at a temperature of 30 °C and a humidity of 70% and incubate it for 14 days.

After fermentation, the culture medium was soaked in methanol/water (*v/v* = 3/1) for 12 h and filtered with diatomaceous earth. The filtrate was vacuum-concentrated at 45 °C to remove methanol. The remaining aqueous phase was extracted with ethyl acetate (4 times) and concentrated to remove ethyl acetate, resulting in crude extract. After three rounds of silica gel column purification (200–300 mesh) and two rounds of C18 column preparation (medium-pressure preparation and high-pressure preparation), pure BKA, iBKA-neo, and iBKA were obtained with purities ranging from 95% to 99%.

### 4.4. Collection of Food Matrices

All samples were fresh, including matrix blanks and samples for detection, which included *Tremella fuciformis* samples, sweet soup dumpling flour samples, rice flour samples, and sour noodles collected from local markets in Beijing. All of the samples were homogenized via the parallel homogenizer mentioned in Section 4.2, dispensed into 50 mL centrifuge tubes and stored at −20 °C until use.

### 4.5. Food Matrices’ Preparation

#### 4.5.1. Direct Extraction Method

For a typical test, homogenized *Tremella fuciformis* samples (1.00 ± 0.01 g) were added to 50 mL centrifuge tubes. A mixed working standard solution was added to the homogenized blank samples and allowed to stand for 10 min. Next, 5 mL of ACN containing 1% HAc was added, followed by 5 min of vortexing and 20 min of ultrasonic extraction. The mixture was subsequently centrifuged at 21,130 rcf for 5 min at 4 °C, after which the supernatants were collected. After this extraction, 1 mL of the supernatant was filtered through a 0.22 μm nylon membrane, after which 50 μL of the mixture was evaporated until dry at 4 °C via a vacuum freeze drier. The residue was redissolved in 50 μL of water/acetonitrile (50:50, *v/v*) solution, vortexed for 5 min, and centrifuged at 21,130 rcf for 5 min to obtain the supernatant before UHPLC‒MS/MS analysis.

#### 4.5.2. Solid-Phase Extraction Method

The process from weighing to filtering the membrane was the same as that described in Section 4.5.1. After filtering, a 1 mL sample was collected and passed through a solid-phase extraction column. The process of solid-phase extraction is as follows (taking the WAX column as an example): Mount the Oasis WAX column to the vacuum extraction unit and set the vacuum to 5″ Hg. The column was then activated with methanol and balanced with water. Next, 1 mL of the prepared sample was passed through a WAX column. Next, 1 mL of 2% FA solution was added as a cleaning solvent. Subsequently, 1 mL of 100% organic eluting solvent (methanol) was added. Finally, 1 mL of a methanol solution with 5% ammonium hydroxide was added as the elution solvent, and all the elution solvents were collected. The subsequent drying and redissolving sample operations were the same as those described in Section 4.5.1.

### 4.6. NMR Measurements

NMR measurements were performed using an AVANCE NEO 400 M spectrometer (Bruker, Switzerland). The acquisition parameters of the hydrogen spectrum were set as follows: ^1^H NMR spectra were measured at 400 MHz, the excitation pulse angle was 30°, the time domain number of data points was 64 k, the scanning width was 8196.7 Hz, the relaxation delay was 1 s, the cumulative sampling time was 16, the probe temperature was 296 K, and the frequency domain number of data points was 64 k. The deuterated methanol (CD_3_OD) solvent peak signal was used as an internal reference, and its chemical shift was set to 3.31 ppm.

The acquisition parameters of the carbon spectrum were set as follows: ^13^C NMR spectra were measured at 101 MHz, the excitation pulse angle was 30°, the time domain number of data points was 64k, the scanning width was 23,809.5 Hz, the relaxation delay was 2 s, the cumulative sampling time was 5000 times, the probe temperature was 296 K, and the frequency domain number of data points was 32k.

For NMR signal assignment, two-dimensional NMR spectra (^1^H-^1^H DQF-COSY, ^1^H-^1^H NOESY, ^1^H-^13^C HSQC, and ^1^H-^13^C HMBC) of the extracts were measured. The acquisition time and delay time of DQF-COSY (double-quantum-filtered chemical shift correlation spectroscopy) are set at 0.2990 s and 1.8894 s, respectively. The spectral widths were 3424.7 Hz (F1) and 3424.7 Hz (F2), and the numbers of data points and scans were 1024 (F1), 128 (F2), and 32, respectively. ^1^H-^1^H NOESY (nuclear Overhauser effect spectroscopy) results were obtained with the following parameters: mixing time of 2.2249 s, relaxation delay of 1.9529 s, and spectral widths of 3424.7 Hz (F1) and 3424.7 Hz (F2), respectively. The number of data points and scanning times were 1024 (F1), 256 (F2), and 8, respectively. The receiver gain was 101.0 and sampling interval was 0.146 s. The ^1^H–^13^C HSQC (heteronuclear single quantum correlation) were generated in phase-sensitive mode with the following acquisition parameters: spectral width, 3424.7 Hz for ^1^H and 16,602.4 Hz for ^13^C; number of data points, 512 for ^1^H and 256 for ^13^C; acquisition time, 0.1495 s; delay time, 1.5 s; and number of scans, 32. The ^1^H–^13^C HMBC (heteronuclear multiple bond correlation) results were measured in absolute mode with the following parameters: spectral width, 3268.0 Hz for ^1^H and 22,137.0 Hz for ^13^C; number of data points, 1024 for ^1^H and 128 for ^13^C; acquisition time, 0.3133 s; delay time, 1.5 s; and number of scans, 32.

NMR signals were analyzed by comparing them with previously published NMR assignments and composition data and by referring to the online NMR database Biological Magnetic Resonance Data Bank (BMRB). The signals were then confirmed and assigned to the candidate compounds based on the ^1^H, ^13^C, and 2D NMR spectra and the results of the spiking experiments.

### 4.7. UHPLC‒MS/MS Analysis

UHPLC‒MS/MS analysis was performed via an ultra-high-performance liquid chromatography−tandem triple quadrupole mass spectrometer (AB Sciex Triple Quad 7500, AB SCIEX, Singapore). Chromatographic separation of the 3 BKAs was carried out with an ACQUITY UPLC BEH C18 column (2.1 mm ×100 mm, 1.7 μm). The mobile phase was composed of solvent A (water with 0.1% formic acid and 2 mmol/L ammonium formate) and solvent B (95% acetonitrile containing 0.1% formic acid and 2 mmol/L ammonium formate). The elution gradient was as follows (Table S3): 0–10.0 min, 43–55% B; 10.0–11.0 min, 55–80% B; 11.0–13.0 min, 80% B; 13.0–13.1 min, 80–43% B; and finally, 13.1–15.0 min, 43% B, at a flow rate of 0.3 mL/min. The temperatures of the column oven and the autosampler were maintained at 40 °C and 10 °C, respectively, throughout their operation. The injection volume was 5 μL.

The MS/MS analysis was performed in both positive and negative ESI modes. The main parameters were optimized as follows: the source temperature was 350 °C, the curtain gas pressure was 40 psi, GAS 1 and GAS 2 were 45 psi and 70 psi, respectively, the ion spray voltage of positive polarity was 5500 V, and the negative polarity was −4500 V. Other parameters, such as retention time (RT), declustering voltage–potential (DP), and collision energy (CE), are shown in Table S2.

### 4.8. Chromatographic and Mass Spectrometry Conditions for HRMS

HRMS coupled with MS Frontiers 8.0 is an efficient tool for identifying the structural and fragmentation patterns of BKAs. The separation of BKAs was achieved with a liquid-phase system, and the column and conditions were the same as those described in Section 4.7. The Orbitrap Exploris 480 HRMS conditions were as follows: the flow rates of the sheath gas, aux gas, and sweep gas were set at 35, 15, and 1 arbitrary unit, respectively. The spray voltages were set at 3.5 kV and −2.5 kV. The mass range (*m/z*) was 100–600. The ion transfer tube temperature and vaporizer temperature were 320 °C and 350 °C, respectively. The mass spectrometer acquisition mode used single-ion measurement (SIM) with data-dependent MS/MS fragmentation (SIM-ddMS2) with a resolution of 60,000 under SIM mode and 30,000 under ddMS2 scanning mode. The normalized collision energies (NCE) were set at 5%, 10%, 15%, and 20%.

### 4.9. Method Validation

This method was validated for its selectivity, LOD, LOQ, matrix effect (ME), linearity, recovery (70–120%), and repeatability (precision, RSD ≤ 20%) according to the Chinese Pharmacopoeia (2020 Edition) and GB5009.189-2023 (National Food Safety Standards). To this end, “blank” food matrices were used, including *Tremella fuciformis*, sweet soup dumpling flour, rice flour, and sour noodles, which were analyzed in triplicate, and BKAs were not detected. Selectivity was assessed in three aspects. First, 24 blank samples were detected to determine if any signals at the same RT as the BKAs were present. Second, the ion ratios of each BKA in different matrices were measured to evaluate the consistency. To do so, the BKAs were simultaneously added to a blank sample at 10 μg/kg in triplicate. The spiked samples were prepared and analyzed via the developed method.

LODs were defined as the lowest spiked concentrations of BKAs in the blank matrix with a signal-to-noise ratio (S/N) of 3. LOQs were defined as the lowest spiked concentrations of BKAs in the blank matrix with a signal-to-noise ratio (S/N) of 10. MEs were evaluated by comparing the response of BKAs in matrix-matched solvents (A) and in water/acetonitrile (50:50, *v/v*) (B) at the same concentrations (1, 5, 10, 50, and 100 ng/mL). The ME was calculated according to the following equation:

$$\mathrm{ME}\left(\%\right)=\left[\frac{\left(A-B\right)}{B}\right]\times 100\%$$

Matrix-matched working solutions at concentrations of 0.05, 0.1, 0.25, 0.5, 1, 2.5, 5, 10, 25, 50, and 100 ng/mL were used to establish matrix-matched calibration curves of the BKAs. Each experiment generated its own standard curve to account for matrix-specific interferences.

The precision of the method was evaluated by performing intraday and interday precision studies, and the results are expressed as the relative standard deviation (RSD). The intraday precision was studied at the same concentrations as those used in the recovery study (LOQ, 5 LOQ, and 10 LOQ), with six replicates for each concentration, whereas the interday precision was evaluated by spiking six samples at the three concentrations on three consecutive days (n = 18).

### 4.10. Statistical Analysis

Count data are expressed as percentages (%), whereas continuous data are presented as the means ± standard deviations. The statistical analysis was performed via the chi-square test, least significant difference test (LSD), and the generalized estimating equation (GEE) method. Partial correlation analysis was conducted to assess the relationships between variables while controlling for the influence of other variables. Each experiment was conducted in triplicate to ensure reliability. Graphs were generated via Origin 2024 and GraphPad Prism 10.0 software.

## Figures and Tables

**Table 1:** Recovery and precision of the developed method at three spiked concentrations in food matrix.

Food Matrix	Compounds	LOD (μg/kg)	LOQ (μg/kg)	Linear Range (μg/kg)	R^2^	Recovery (n = 6)			Intraday RSD (n = 6)			Interday RSD (n = 18)		
						LOQ	5 LOQ	10 LOQ	LOQ	5 LOQ	10 LOQ	LOQ	5 LOQ	10 LOQ
Tremella fuciformis (TF)	Bongkrekic Acid	0.08	0.25	0.25–500	0.9958	114.35%	108.41%	106.13%	2.13%	1.74%	1.24%	4.09%	2.44%	2.18%
	Isobongkrekic Acid-neo	0.08	0.25	0.25–500	0.9956	112.56%	108.82%	105.27%	4.20%	3.08%	2.30%	9.52%	2.85%	2.80%
	Isobongkrekic Acid	0.08	0.25	0.25–500	0.9968	105.18%	101.78%	94.64%	8.58%	2.62%	1.82%	8.40%	2.05%	4.32%
Sweet soup dumpling flour (SSDF)	Bongkrekic Acid	0.08	0.25	0.25–500	0.9949	104.78%	97.49%	101.76%	5.10%	1.68%	2.67%	3.92%	4.92%	6.05%
	Isobongkrekic Acid-neo	0.08	0.25	0.25–500	0.9925	110.69%	102.32%	104.85%	4.30%	1.34%	1.71%	5.34%	3.03%	3.44%
	Isobongkrekic Acid	0.08	0.25	0.25–500	0.9976	100.72%	86.47%	90.89%	5.31%	3.34%	1.24%	3.23%	6.72%	8.55%
Rice flour (RF)	Bongkrekic Acid	0.08	0.25	0.25–500	0.9996	111.78%	102.96%	91.62%	3.40%	1.15%	1.89%	0.72%	8.00%	12.67%
	Isobongkrekic Acid-neo	0.08	0.25	0.25–500	0.9990	114.84%	104.35%	98.90%	2.10%	4.31%	6.27%	3.97%	6.25%	9.37%
	Isobongkrekic Acid	0.08	0.25	0.25–500	0.9986	95.68%	100.87%	93.14%	4.19%	2.40%	1.56%	6.77%	7.37%	9.31%
Sour noodles (SN)	Bongkrekic Acid	0.08	0.25	0.25–500	0.9991	92.47%	87.96%	84.41%	2.48%	1.72%	2.15%	9.60%	10.46%	11.66%
	Isobongkrekic Acid-neo	0.08	0.25	0.25–500	0.9990	91.91%	90.08%	86.99%	3.47%	2.32%	3.50%	5.22%	6.69%	6.08%
	Isobongkrekic Acid	0.08	0.25	0.25–500	0.9985	97.81%	85.49%	82.32%	2.96%	3.45%	2.24%	6.35%	7.34%	8.84%

## Data Availability

The original contributions presented in this study are included in the article/Supplementary Material. Further inquiries can be directed to the corresponding authors.

## Supplementary Materials

The following supporting information can be downloaded at: https://www.mdpi.com/article/10.3390/toxins17050223/s1, Figure S1: The structural identification of BKA; Figure S2. Characteristics and fragmentation patterns detected by negative ion mass spectrometry for iBKA and iBKA-neo; Figure S3. Characteristics and fragmentation patterns detected by positive ion mass spectrometry for iBKA and iBKA-neo; Figure S4. iBKA-neo and iBKA linear ranges; Figure S5. Optimization of the chromatographic separation method for BKA isomers (PBT column); Figure S6. Optimization of extraction and enrichment methods for BKA isomers; Table S1: Detailed information of BKA isomers; Table S2. The mass parameters of BKA isomers; Table S3. Gradient elution program for BKA isomers−ACQUITY UPLC BEH C18 column; Table S4. Gradient elution program for BKA isomers−DCpak PBT column.
