# Supplementary material for: Bongkrekic Acid and Its Novel Isomers: Separation, Identification, and Determination in Food Matrices
Source: Toxins (Basel). 2025 May 2;17(5):223. doi: 10.3390/toxins17050223 (PMC12115625; doi:10.3390/toxins17050223)
Supplement: Supplementary file 1 [file toxins-17-00223-s001.zip › toxins-3593433-supplementary.pdf]

Supplementary Materials

Bongkrekic Acid and Its Novel Isomers: Separation, Identification, and Determination in Food Matrices

Suhe Dong, Danli Liu, Runfeng Lin, Yingjie Zhu, Peihong Zhu, Xin Jiang, Jie Mao, Yanqing Cao, Jing Peng, Tianyue Zhao, Danning Shen, Tao Li, Kun He*, Na Wang*

National Center of Biomedical Analysis, Beijing 100039, China

*Corresponding author:
Kun He, Email: hk@proteomics.cn
Na Wang, Email: nwang@ncba.ac.cn

Supplementary Figures

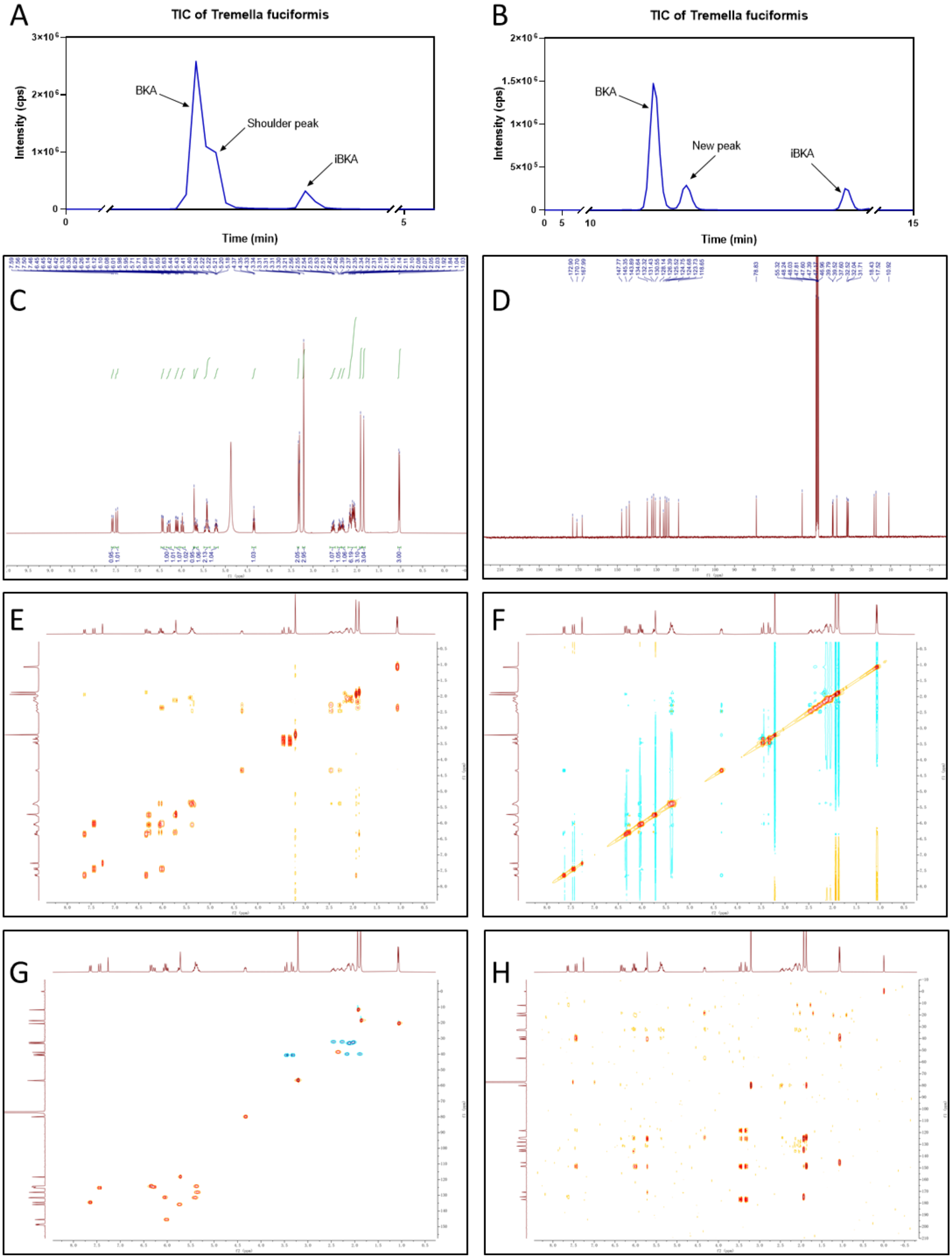

Figure S1. The structural identification of BKA. (A) TIC of BKAs in *Tremella fuciformis* infected with *Burkholderia gladioli* according to established literature protocol. **(B)** TIC of BKAs in *Tremella fuciformis* infected with *Burkholderia gladioli* according to optimized protocol. **(C)** ^{1}H NMR of BKA (400 MHz, CD_3OD). **(D)** ^{13}C NMR of BKA (101 MHz, CD_3OD). **(E)** ^{1}H-^{1}H DQF-COSY of BKA (F1, 3424.7 Hz; F2, 3424.7 Hz, CD_3OD). **(F)** ^{1}H-^{1}H NOESY of BKA (F1, 3424.7 Hz; F2, 3424.7 Hz, CD_3OD). **(G)** ^{1}H-^{13}C HSQC of BKA (^{1}H 3424.7 Hz, ^{13}C 16602.4 Hz). **(H)** ^{1}H-^{13}C HMBC of BKA (^{1}H 3268.0 Hz, ^{13}C 22137.0

Hz). In detail, ^{1}H NMR (400 MHz, CD_3OD): δ 7.57 (d, J = 12.0 Hz, 1H), 7.48 (d, J = 16.1 Hz, 1H), 6.43 (dd, J = 12.0, 1.7 Hz, 1H), 6.29 (dd, J = 15.1, 10.9 Hz, 1H), 6.11 (dd, J = 16.1, 7.5 Hz, 1H), 5.98 (t, J = 10.9 Hz, 1H), 5.71 (s, 1H), 5.67 (dt, J = 14.9, 7.1 Hz, 2H), 5.52-5.36 (m, 1H), 5.52-5.36 (m, 1H), 5.21 (dt, J = 10.9, 7.6 Hz, 1H), 3.34 (s, 2H), 3.21 (s, 3H), 4.35 (t, J = 7.1 Hz, 1H), 2.62-2.49 (m, 1H), 2.44-2.36 (m, 1H), 2.35-2.28 (m, 1H), 2.20-1.98 (m, 2H), 2.20-1.98 (m, 2H), 2.20-1.98 (m, 2H), 1.92 (s, 3H), 1.84 (s, 3H), 1.03 (d, J = 6.7 Hz, 3H). ^{13}C NMR (101 MHz, CD_3OD):δ 132.32, 124.75, 124.68, 125.52, 143.89, 130.55, 118.65, 134.64, 128.14, 131.43, 123.73, 39.79, 55.32, 78.83, 31.71, 37.60, 39.52, 32.52, 32.04, 10.92, 17.52, 18.43, 172.90, 167.99, 147.77, 145.35, 126.39, 170.70.

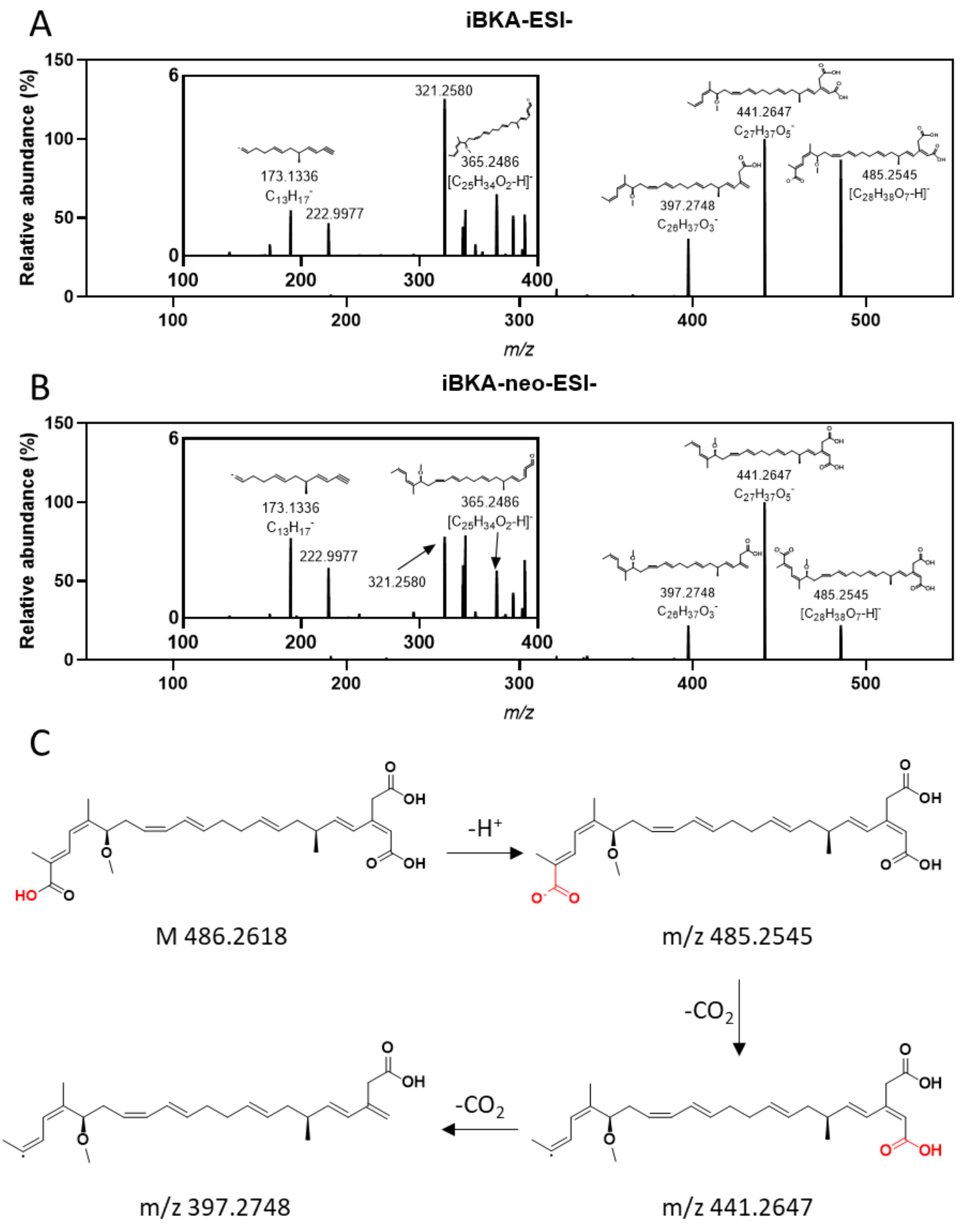

Figure S2. Characteristics and fragmentation patterns detected by negative ion mass spectrometry for iBKA and iBKA-neo. (A) Fragment ion characteristics of iBKA in ESI negative mode $[M\text{-}H]^-$, high-resolution mass spectrometry in SIM-ddMS2 detection mode, and HCD (%) steps of 5, 10, 15, and 20 for normalization. **(B)** Fragment ion characteristics of iBKA-neo in ESI negative mode $[M\text{-}H]^-$, high-resolution mass spectrometry SIM-ddMS2 detection mode, and HCD (%) steps of 5, 10, 15, and 20 for normalization. **(C)** The fragmentation law of the main product ions of BKA in ESI negative mode (MS Frontiers 8.0).

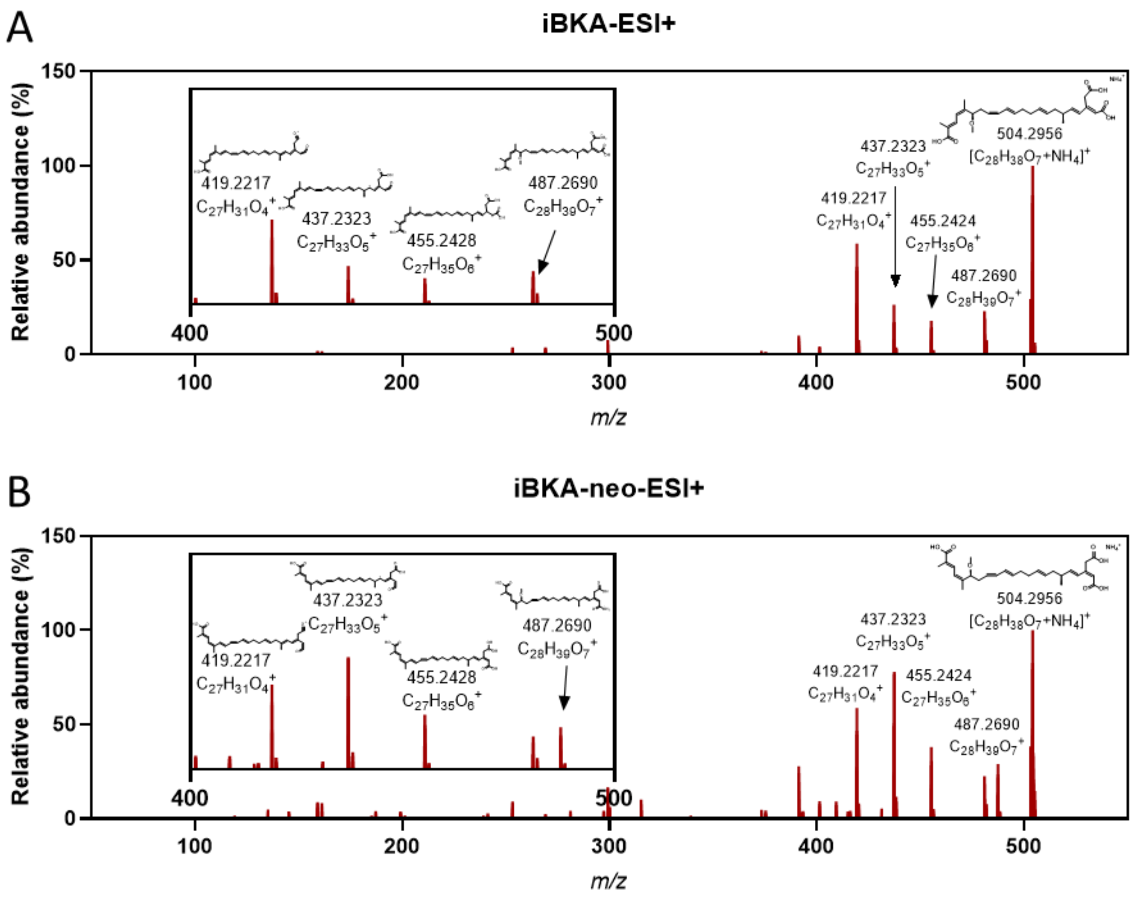

Figure S3. Characteristics and fragmentation patterns detected by positive ion mass spectrometry for iBKA and iBKA-neo. (A) Fragment ions characteristics of iBKA in ESI positive mode $[M+NH_4]^+$, high-resolution mass spectrometry (SIM-ddMS2) detection mode, and HCD (%) steps of 5, 10, 15, and 20 for normalization. **(B)** Fragment ions characteristics of iBKA-neo in ESI positive mode $[M+NH_4]^+$, high-resolution mass spectrometry (SIM-ddMS2) detection mode, and HCD (%) steps of 5, 10, 15, and 20 for normalization.

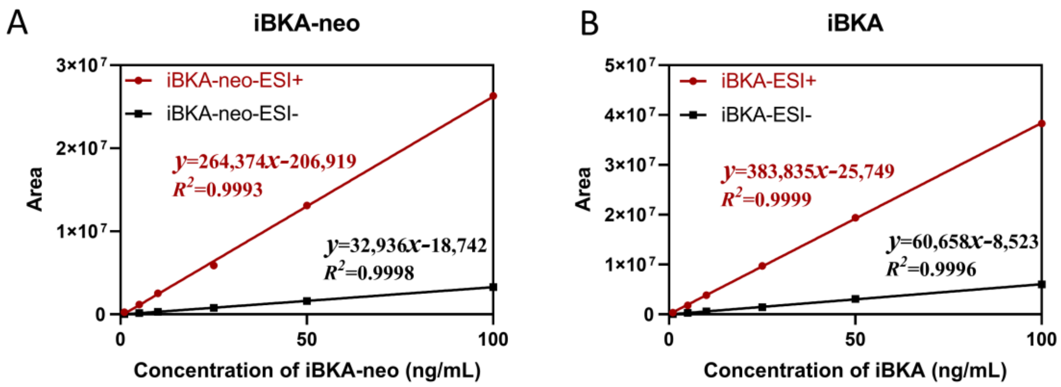

Figure S4. iBKA-neo and iBKA linear ranges. Linear range and correlation coefficient of the mass spectrometry response of **(A)** iBKA-neo and **(B)** iBKA detected in ESI positive and negative mode.

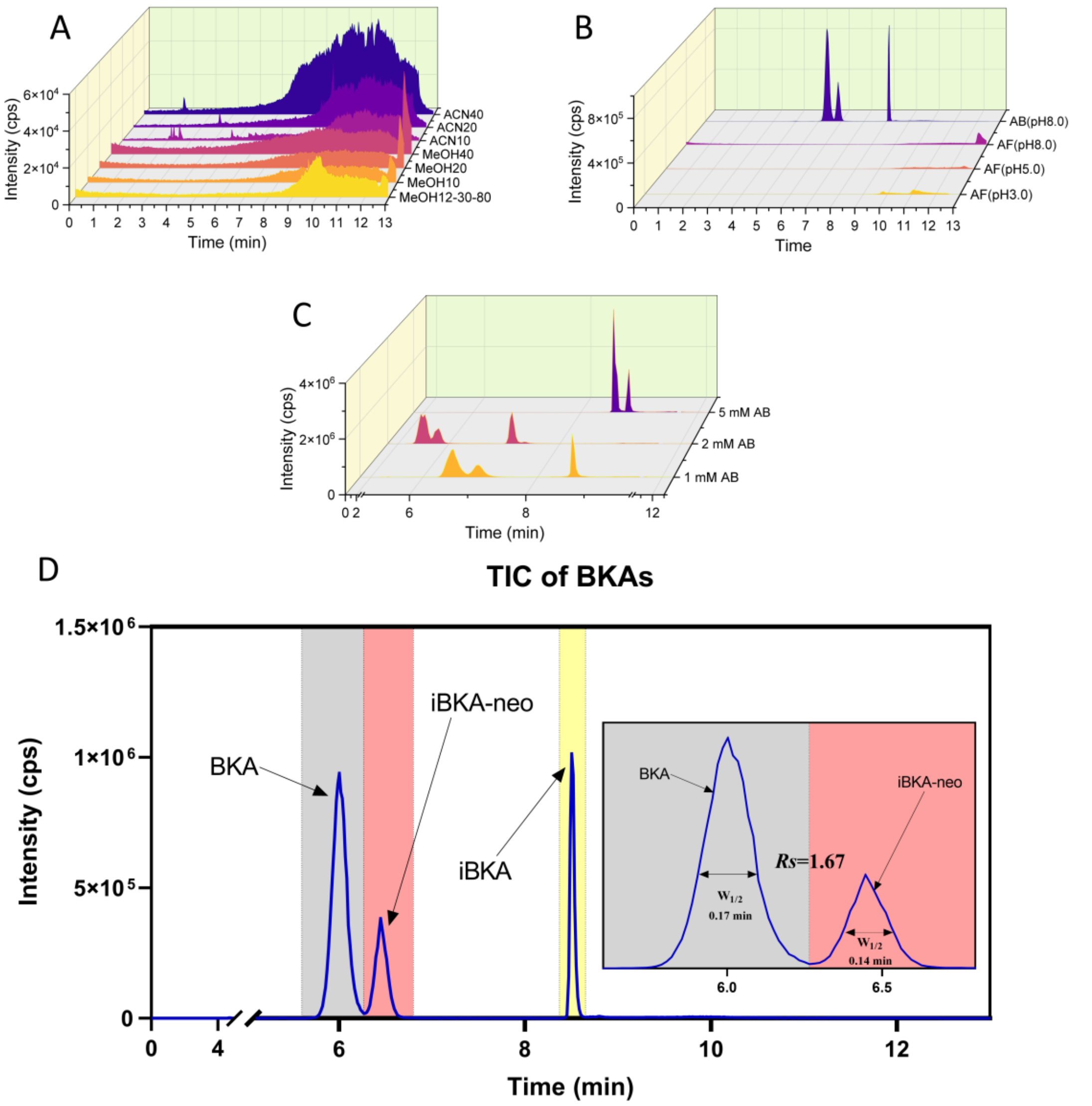

Figure S5. Optimization of the chromatographic separation method for BKA isomers (PBT column). (A) TICs of the separation efficiency of BKA isomers by MEOH and ACN as organic phases with initial mobile phases of 20%, 40%, 60%, and MEOH segmented gradient changes of 12%−30%−80%. **(B)** TICs of the separation efficiency of BKA isomers in the mobile phase buffer salt system at various pH (AF, pH 3.0; AF, pH 5.0; AF, pH 8.0; AB, pH 8.0). **(C)** TICs of the separation efficiency of different concentrations of AB (1 mM, 2 mM, and 5 mM) on BKA isomers in the mobile phase. **(D)** TIC of the separation efficiency of BKA isomers optimized by chromatographic conditions. $W_{1/2}$ represents the full width at half maximum, Rs represents the separation degree.

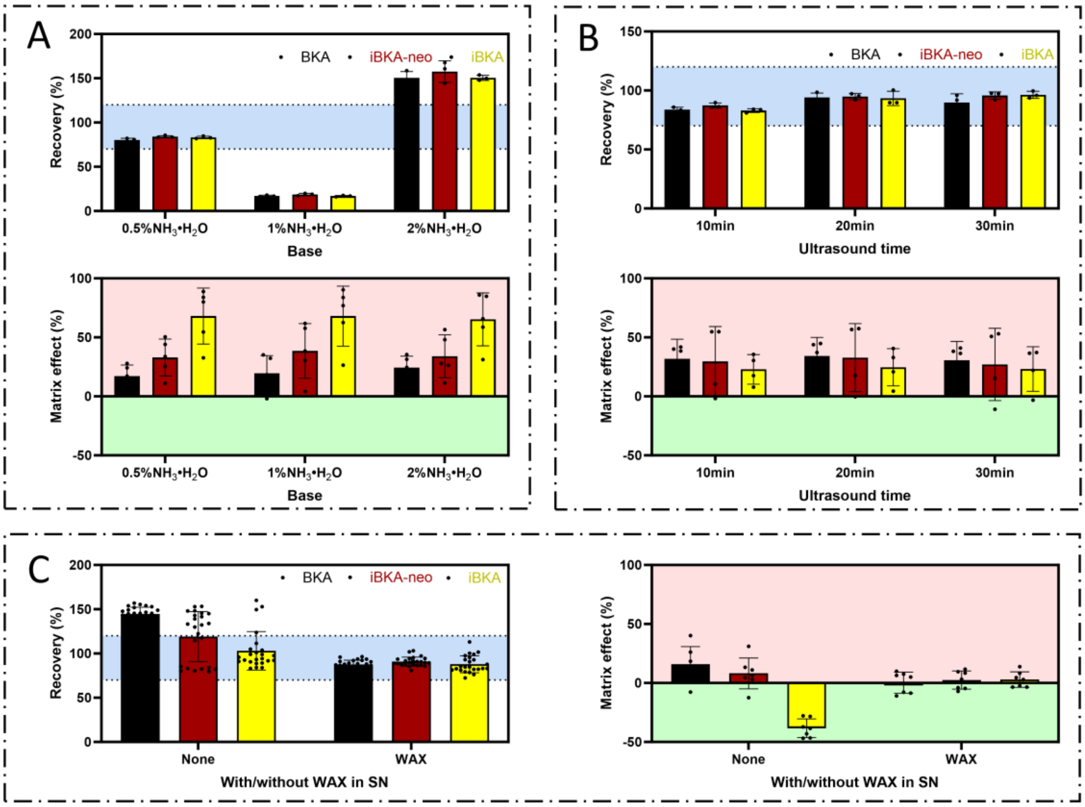

Figure S6. Optimization of extraction and enrichment methods for BKA isomers. (A) Effects of different ratios of NH_3•H_2O (0.5%, 1%, and 2%) in the extraction reagent on the extraction recovery rates and matrix effects of BKA isomers in the *Tremella fuciformis* matrix. **(B)** Effects of different ultrasonication times (10 min, 20 min, and 30 min) on the extraction recovery rates and matrix effects of BKA isomers in the *Tremella fuciformis matrix*. **(C)** Influence of the WAX SPE column on the recovery rates and matrix effects of BKA isomers in the sour noodles matrix.

Table S1. Detailed information of BKA isomers

Compounds	Molecule Formula	LogP	Solvent (100 μg/mL)
Bongkrekic Acid	$C_{28}H_{38}O_7$	5.8856	ACN: H_2O=1:1
Isobongkrekic Acid-neo	$C_{28}H_{38}O_7$	5.8856	ACN: H_2O=1:1
Isobongkrekic Acid	$C_{28}H_{38}O_7$	5.8856	ACN: H_2O=1:1

Table S2. The mass parameters of BKA isomers

Compounds	Precursor ion (m/z)	Product ion (m/z)	Ionization	EP (V)	CE (V)	CXP (V)
Bongkrekic Acid	504	419	$[M+NH_4]+$	10	15	13
	504	437	$[M+NH_4]+$	10	12	13
Isobongkrekic Acid-neo	504	437	$[M+NH_4]+$	10	12	12
	504	419	$[M+NH_4]+$	10	15	12
Isobongkrekic Acid	504	419	$[M+NH_4]+$	10	15	11
	504	437	$[M+NH_4]+$	10	11	13
Bongkrekic Acid	485	441	[M-H]-	-10	-15	-12
	485	397	[M-H]-	-10	-25	-11
Isobongkrekic Acid-neo	485	397	[M-H]-	-10	-25	-10
	485	441	[M-H]-	-10	-15	-11
Isobongkrekic Acid	485	441	[M-H]-	-10	-19	-10
	485	397	[M-H]-	-10	-25	-10

Table S3. Gradient elution program for BKA isomers–ACQUITY UPLC BEH C18 column

Time	Flow rate (mL/min)	A (%)	B (%)
0	0.3	57	43
10	0.3	45	55
11	0.3	20	80
13	0.3	20	80
13.1	0.3	57	43
15	0.3	57	43

Table S4. Gradient elution program for BKA isomers−DCpak PBT column

Time	Flow rate (mL/min)	A (%)	B (%)
0	0.4	88	12
6	0.4	70	30
8	0.4	20	80
10.5	0.4	20	80
10.6	0.4	88	12
13	0.4	88	12
